# The roles and mechanisms of endoplasmic reticulum stress-mediated autophagy in animal viral infections

**DOI:** 10.1186/s13567-024-01360-4

**Published:** 2024-09-03

**Authors:** Lan Chen, Miaozhan Wei, Bijun Zhou, Kaigong Wang, Erpeng Zhu, Zhentao Cheng

**Affiliations:** 1 Department of Veterinary Medicine, College of Animal Science, Guizhou University, Guiyang, 550025 China; 2 Key Laboratory of Animal Disease and Veterinary Public Health of Guizhou Province, College of Animal Science, Guizhou University, Guiyang, 550025 China

**Keywords:** Endoplasmic reticulum stress (ERS), cellular unfolded protein response (UPR), autophagy, animal viruses, immune escape

## Abstract

The endoplasmic reticulum (ER) is a unique organelle responsible for protein synthesis and processing, lipid synthesis in eukaryotic cells, and the replication of many animal viruses is closely related to ER. A considerable number of viral proteins are synthesised during viral infection, resulting in the accumulation of unfolded and misfolded proteins in ER, which in turn induces endoplasmic reticulum stress (ERS). ERS further drives three signalling pathways (PERK, IRE1, and ATF6) of the cellular unfolded protein response (UPR) to respond to the ERS. In numerous studies, ERS has been shown to mediate autophagy, a highly conserved cellular degradation mechanism to maintain cellular homeostasis in eukaryotic cells, through the UPR to restore ER homeostasis. ERS-mediated autophagy is closely linked to the occurrence and development of numerous viral diseases in animals. Host cells can inhibit viral replication by regulating ERS-mediated autophagy, restoring the ER's normal physiological process. Conversely, many viruses have evolved strategies to exploit ERS-mediated autophagy to achieve immune escape. These strategies include the regulation of PERK-eIF2α-Beclin1, PERK-eIF2α-ATF4-ATG12, IRE1α-JNK-Beclin1, and other signalling pathways, which provide favourable conditions for the replication of animal viruses in host cells. The ERS-mediated autophagy pathway has become a hot topic in animal virological research. This article reviews the most recent research regarding the regulatory functions of ERS-mediated autophagy pathways in animal viral infections, emphasising the underlying mechanisms in the context of different viral infections. Furthermore, it considers the future direction and challenges in the development of ERS-mediated autophagy targeting strategies for combating animal viral diseases, which will contribute to unveiling their pathogenic mechanism from a new perspective and provide a scientific reference for the discovery and development of new antiviral drugs and preventive strategies.

## Introduction

The endoplasmic reticulum (ER) is a cytoplasmic membranous organelle in mammalian cells. It is the major site of synthesis, folding, maturation, and transport of most intracellular secretory and membrane proteins and Ca^2+^ storage. ER homeostasis is also an important safeguard for maintaining normal cellular activities [1–3]. Physiological dysfunction in the ER occurs when cells are exposed to stimuli such as hypoxia, calcium overload, free radical attack, and microbial infection. ER dysfunction results in the accumulation of misfolded or unfolded proteins in the ER lumen, which can cause an imbalance in calcium homeostasis and trigger endoplasmic reticulum stress (ERS) [4–6].

The cells experiencing ERS can trigger an evolutionarily conserved adaptive mechanism, unfolded protein response (UPR), to restore normal ER function [7]. The basic UPR mechanism in mammals is initiated by three ER transmembrane protein sensors, including protein kinase RNA-activated (PKR)-like ER resident kinase (PERK), inositol-requiring enzyme-1 (IRE1), and activating transcription factor-6 (ATF6). These sensors regulate diverse signalling pathways to inhibit misfolded protein synthesis, enhance misfolded protein degradation, and promote correct folding of unfolded proteins. Such a regulation alleviates ERS [8, 9].

ERS and autophagy are two evolutionarily conserved cellular activities in eukaryotic cells that can perform their roles independently. However, they can also be linked and share some common functions, including initiating intracellular degradation pathways, removing stress signals, and restoring intracellular homeostasis [10].

Autophagy is regulated through various cellular signalling pathways, and recently, in addition to the classical autophagy-inducing pathway, ERS has been recognised as one of the significant pathways for regulating cellular autophagy [11, 12]. Growing evidence has demonstrated the significant roles of ERS-mediated autophagy in restoring ER and intracellular homeostasis. Furthermore, ERS-mediated autophagy is closely linked to the developmental process of a wide range of viral diseases in animals. Animal viruses, such as Japanese encephalitis virus (JEV), classical swine fever virus (CSFV), and porcine epidemic diarrhoea virus (PEDV), can induce ERS. In turn, this can trigger autophagy by activating the cellular UPR pathways [13–16]. However, the involvement of UPR pathways and UPR-related signalling molecules in regulating autophagy and their effects on viral replication depends on the specific animal viruses and their cellular contexts [17, 18]. The relationships and mechanisms of action between cellular ERS-mediated autophagy pathways and animal viruses are complex and still not fully understood.

In this paper, we examine the roles and mechanisms of the ERS-mediated autophagy pathways in different animal viral infections and consider the future directions and challenges for developing ERS-mediated autophagy targeting strategies to combat these diseases. These latest research advances will aid in uncovering the mystery surrounding complex host-virus interactions during animal viral infections. They will serve as scientific references for developing new anti-animal viral drugs targeting ERS-mediated autophagy pathways. Moreover, they will contribute to developing effective strategies for preventive therapy in clinical practices.

## ERS-driven UPR in response to animal viral infections

As mentioned, ERS can be induced by certain factors and stimuli [4–6]. The cells undergoing ERS initiate the UPR to restore ER homeostasis and achieve cell survival [6, 19, 20]. The UPR signalling pathway is primarily mediated by the ER sensors and molecular chaperone glucose-regulated protein 78 (GRP78). This chaperone is also known as immunoglobulin heavy chain binding protein (BiP) [21]. Three UPR signalling pathways have been identified and named after three ER membrane-sensing proteins: PERK, ATF6, and IRE1 [22]. Under normal physiological conditions, BiP binds to these three sensor proteins and inhibits their activity. When cells experience ERS, sensor proteins dissociate from BiP and become activated, initiating a cascade response that involves UPR pathways to restore ER homeostasis [23]Typically, the PERK pathway is the signalling pathway that is preferentially activated after the onset of ERS [24].

When PERK is phosphorylated and dimerised, it directly phosphorylates eukaryotic initiation factor 2α (eIF2α), resulting in a widespread decrease in intracellular protein translation. It selectively enhances the translation of activating transcription factor 4 (ATF4) and C/EBP homologous protein (CHOP). These two proteins serve as a feedback mechanism to restore cellular protein synthesis [25, 26]. Additionally, they can restore ER homeostasis by regulating the expression of genes, such as ER chaperones and redox regulators [27].

In cases where cells undergo excessive ERS, CHOP may trigger apoptosis [28]. IRE1 is often considered the most evolutionarily conserved ERS sensor, possessing both RNAase and kinase activities [19]. Once activated, IRE1 undergoes dimerisation and autophosphorylation, initiating its own RNAase activity to specifically remove a 26-base intron from the X-box binding protein 1 (XBP1) mRNA. This activation results in the production of the active transcription factor XBP1s, which translocates to the nucleus and regulates the expression of UPR genes, thereby promoting proper protein folding in the ER and ER-associated degradation (ERAD) [29, 30]. IRE1 can also cause the degradation of various mRNAs within the ER through regulated IRE1-dependent decay (RIDD) [31]. Furthermore, IRE1 has been shown to promote ERS-mediated apoptosis through activation of c-Jun N-terminal kinase (JNK) and cysteinyl aspartate specific proteinase 12 (caspase12) [32]. In the ATF6 pathway, after ATF6 is cleaved by S1P and S2P proteases on the Golgi apparatus, the activated ATF6(N) enters the nucleus. It then enhances the expression of ER protein chaperone molecules (e.g. BiP), XBP1*,* CHOP and ERAD components. This process promotes the correct folding and trafficking of unfolded or misfolded proteins, ultimately mitigating ERS and maintaining normal ER function [33]. Collectively, the three branches of the UPR are cross-linked. Together, they form a complex signal network [34]. However, in some cases, the UPR can lead to cellular dysfunction and, ultimately, cell death when cells are exposed to overintense or prolonged ERS [35].

Numerous studies have shown that the UPR plays a significant role in the development of viral diseases in animals. Hosts can activate the cellular UPR mechanism to resist viral infection, while viruses can manipulate the UPR to facilitate their replication and infection. Essentially, the UPR mechanism is a double-edged sword in the battle between animal viruses and host cells.

### DNA viruses associated with ERS

ERS-driven UPR represents distinctive roles and mechanisms in different DNA virus infections. Porcine circovirus 2 (PCV2) can activate one or three UPR signalling pathways upon infection or protein transfection. It has been reported that PCV2 infection of both porcine kidney 15 (PK-15) cells and porcine alveolar macrophage (PAM) cells up-regulated BiP and selectively activate the PERK pathway to enhance PCV2 replication. However, neither the ATF6 nor IRE1 pathways are activated [36]. Interestingly, the PCV2 ORF5 protein can increase the phosphorylation levels of PERK and eIF2α and up-regulate the expression of ATF4 in PAM cells. Moreover, ORF5 increases the phosphorylation of IRE1 to promote the splicing of XBP1 and induces the splicing of ATF6 to promote PCV2 replication [37]. To better understand this discrepancy, we need to investigate whether it is due to differences in strains or other factors.

It is puzzling that co-infection with PCV2 and pseudorabies virus (PRV) has been found to induce ERS and activate the PERK-eIF2α-ATF4-CHOP and IRE1-XBP1 pathways instead of the ATF6 pathway [38]. Such an effect suggests that viral co-infection exhibits differences from a single viral infection in the mechanisms of ERS-driven UPR activation. ERS caused by bovine herpesvirus-1 (BoHV-1) infection of Madin-Darby bovine kidney (MDBK) cells can activate all three UPR sensors, and upon further study, it was found that the ATF6 pathway does not affect viral replication.

However, the replication of BoHV-1 is negatively regulated by the knockdown of PERK and IRE1 using GSK2606414 (PERK inhibitor) and 4μ8C (IRE1 inhibitor). This negative regulation suggests that BoHV-1-induced PERK and IRE1 pathways may promote viral replication [39]. In addition, infection with the chikungunya virus (CHKV) can trigger activation of the IRE1 and ATF6 pathways while the PERK pathway is inhibited. Further study found that human embryonic kidney 293 (HEK293) cells treated with 3-ethoxy-5,6-dibromosalicylaldehyde (IRE1 inhibitor) and AEBSF (ATF6 inhibitor) significantly inhibits viral replication [40], suggesting that the positive role of CHKV-activated IRE1 and ATF6 pathways in CHKV replication.

Some DNA viruses, such as PRV, duck enteritis virus (DEV), and Marek's disease virus (MDV), can only regulate two of the UPR signalling pathways after infection. However, each virus has unique mechanisms for doing so. PRV and DEV infection up-regulate BiP expression, activating the PERK and IRE1 pathways rather than the ATF6 pathway. PRV activates the PERK pathway with up-regulation of ATF4 and CHOP. This activation simultaneously triggers the splicing of XBP1 mRNA, while ERS, induced by thapsigargin, a widely used ERS agonist, promotes PRV replication in suspension-cultured baby hamster kidney-21 (BHK-21) cells [41, 42]. However, the IRE1-XBP1 pathway activated by PRV infection does not have a significant effect on the replication of PRV [43]. DEV-induced ERS causes ER expansion, but the role of specific signalling molecules of UPR on viral replication is not reflected in the study [44]. Although the exact mechanism is still unknown, MDV also primarily activates the IRE1 and ATF6 pathways of the UPR but not the PERK pathway [45].

In addition, certain DNA viruses only regulate one of the UPR signalling pathways after infection. Porcine parvovirus (PPV) infection activates the PERK-mediated UPR, which significantly prevents PPV replication. CHOP has also been identified as a key factor in inhibiting PPV replication, while PPV-induced UPR further inhibits viral replication by promoting apoptosis [46]. It has also been found that mouse cytomegalovirus (MCMV) infection inhibits IRE1-mediated mRNA splicing and the expression of XBP1s to promote MCMV replication [47]. The ERS-driven UPR collectively plays a crucial role in the replication and infection of animal DNA viruses. However, the specific processes involved may vary.

### RNA viruses associated with ERS

The largest area in the intracellular membrane is the ER membrane, and RNA viruses proliferate on the inner membrane of the host cell. Therefore, the replication of RNA viruses is often associated with ER [48, 49]. When large numbers of viral proteins accumulate ER, ERS is probable. Most RNA viruses can hold ERS to evade the antiviral mechanism of host cells, such as PEDV, Zika virus (ZIKV), Tembusu virus (TMUV), CSFV, and porcine deltacoronavirus (PDCoV), all of which can regulate three UPR signalling pathways upon viral infections. PEDV infection up-regulates BiP expression and enhances the phosphorylation of PERK, eIF2α, and IRE1. It also induces ATF6 cleavage [14, 50, 51].

The CH/SXYL/2016 variant strain of PEDV infection induces autophagy to promote viral replication via PERK and IRE1 [14]. However, when treated with 2-Deoxy-D-glucose, an ERS agonist, it activates UPR while limiting the proliferation of PEDV strain HLJBY [51]. It remains unclear whether the promoting or inhibiting effect of the ERS-driven UPR on PEDV replication is specific to certain strains of PEDV. Furthermore, certain PEDV proteins have been found to result in ERS. Specifically, the PEDV E protein is capable of inducing ERS to facilitate continual replication of PEDV [52].

A recent study found that the PEDV Nsp14 protein down-regulates BiP expression, which can inhibit PEDV replication. This finding suggests that PEDV may evade the inhibitory effect of ERS by inhibiting BiP, thereby promoting its own replication [53]. When the African green monkey kidney cell line Vero is transfected with PEDV S protein, BiP expression increases, and the PERK pathway is activated. However, viral replication is suppressed by using Salubrinal (a selective inhibitor of eIF2α dephosphorylation) [54], suggesting that ERS induced by PEDV S protein negatively regulates PEDV replication.

The exact processes of ERS caused by other PEDV proteins or their effects on viral replication are still uncertain and require further investigation, such as the PEDV N protein, the Nsp6 protein, and the ORF3 protein, although all of them can up-regulate BiP expression [55–57]. For instance, the PEDV N protein activates NF-κB to induce ERS [56], but the precise mechanism is unknown. PEDV ORF3 protein induces ERS by activating the PERK-eIF2α signalling pathway, but its effect on viral replication has not been determined [57]. Moreover, a recent study found that BiP is slightly up-regulated 24 h after ZIKV infection of human choriocarcinoma (JEG) cells and that the up-regulation of BiP expression contributes to maintaining ER homeostasis. However, a BiP increase is not observed in ZIKV-infected human choriocarcinoma (JAR) cells and human villous trophoblasts (HTR-8) cells [58].

Additionally, when mouse neuronal cells are infected with ZIKV, it triggers the splicing of XBP1 and its nuclear translocation. Similarly, it induces the hydrolysis of ATF6 protein and the nuclear translocation of ATF6(N) both in vitro and in vivo, thereby contributing to viral replication. However, ZIKV infection significantly increases eIF2α phosphorylation, which does not affect ZIKV replication [59, 60].

TMUV, which belongs to the same family as ZIKV, has been shown to decrease egg production and cause neurological issues in birds. Additionally, in infected BHK-21 cells, it up-regulates the expression of BiP and GRP94. The PERK pathway is activated early in TMUV infection, leading to up-regulation of ATF4 and CHOP. The IRE1 pathway is also activated, resulting in the splicing of XBP1 mRNA. Increased expression of ATF6 and activity of ERS response elements suggest that the ATF6 pathway is also activated during TMUV infection. In addition, the levels of BiP and XBP1s are significantly elevated in TMUV-infected Chicken embryo fibroblast cell lines DF-1 [61], suggesting that the mechanism of UPR activation by TMUV infection may be cell-dependent. CSFV infection activates the IRE1 pathway and eIF2α-ATF4-CHOP signalling of the PERK pathway [15, 62, 63]. However, the ATF6 pathway can be activated by CSFV infection of porcine testicular (ST) cells in vitro and in vivo [15, 62].

Another report suggests that CSFV infection slightly inhibits the ATF6 pathway in PK-15 cells [63] and that CSFV-induced activation of UPR, particularly the IRE1 branch, favours CSFV replication [63]. Furthermore, the same effect can be achieved by expressing the CSFV NS5A protein alone, which has been shown to activate UPR and promote CSFV replication [62]. Therefore, the effect of UPR in PDCoV infection has been further examined after the demonstration of activation of the IRE1-XBP1 pathway, ATF6 pathway, and PERK-eIF2α pathway. The treatment with ISRIB (a PERK-specific inhibitor) was found to promote PDCoV replication; however, the IRE1 pathway was shown to have no effect on PDCoV replication. Interestingly, inhibition of ATF6 significantly inhibits the mRNA expression of BiP and GRP94, thus inhibiting PDCoV replication [64]. These results indicate that the activation of UPR by PDCoV plays diverse regulatory roles in viral replication.

Some RNA viruses can regulate two of the UPR signalling pathways after infection, such as tick-borne encephalitis virus (TBEV), porcine reproductive and respiratory syndrome virus (PRRSV), and JEV. TBEV infection with Vero E6 cells causes the activation of IRE1 and ATF6 pathways. This activation leads to mRNA and protein expression of spliced XBP1s, translocation of ATF6, and expression of ATF6(N) [65]. The pretreatment of cells with 3,5-dibromosalicylaldehyde (an IRE1 inhibitor) and taurodeoxycholic acid (TUDCA, an ERS inhibitor) prior to viral infection shows that TBEV replication is significantly restricted [65]. This outcome suggests that TBEV-induced ERS is beneficial for viral replication. Notably, the mechanism through which the same virus induces UPR and its regulatory role in viral replication may differ. For example, this is observed with PRRSV and JEV. Monkey embryonic kidney epithelial (MARC-145) cells infected by PRRSV strain WUH3, Chinese highly pathogenic PRRSV strain JXwn06, and low pathogenic PRRSV strain HB1/3.9 induce BiP expression and activate the PERK and IRE1 pathways to promote viral replication. This finding is shown by the increased phosphorylation levels of PERK and IRE1 and the specific cleavage of XBP1 mRNA [66, 67].

Likewise, another study indicated that the PRRSV strain VR2385 induced UPR activation through all three branches and effectively suppressed viral replication after treating cells with chemical ERS inducers [68]. This outcome suggests that PRRSV-induced ERS is deleterious to its own viral replication. The reasons for this discrepancy may be related to the specificity of the viral strains, but further investigation is needed.

When the mouse microglia cell line BV2 cells are infected with JEV, BiP and ATF4 mRNA levels are increased. This increase also leads to the promotion of eIF2α phosphorylation and accumulation of XBP1s mRNA, indicating that the PERK and IRE1 pathways are activated rather than the ATF6 signalling pathway [69]. In contrast, when the human neuroblastoma cell line SH-SY5Y is infected with JEV, the PERK expression level increases while no significant changes are observed in the expression of IRE1 and ATF6 [70]. The levels of BiP mRNA and protein increase significantly after JEV infection in murine neuroblastoma cells (Neuro-2α) and BHK-21 cells. This suggests an induction of the ERS, but the specific UPR pathway is yet to be determined [69]. Treatment with 4-PBA (an ERS inhibitor) suppresses JEV replication in BV2 cells [71], inferring that ERS plays a positive role in JEV replication.

After an infection, certain RNA viruses, such as the peste des petits ruminants virus (PPRV) and the Newcastle Disease virus (NDV), have the ability to regulate one of the UPR signalling pathways. PPRV infection increases BiP expression and, therefore, promotes the phosphorylation levels of PERK and eIF2α proteins and the mRNA expression levels of ATF4 and CHOP. In contrast, neither ATF6 nor IRE1 pathways are activated, suggesting that the PERK pathway is mainly responsible for PPRV-induced ERS. Moreover, inhibition of the PERK pathway by GSK or PERK-interfering agents reduces PPRV replication, suggesting that PPRV can utilise the PERK pathway to promote its replication [72].

NDV infection of chicken embryo fibroblasts reveals ERS-induced BiP overexpression, indicating the occurrence of ERS [73]. Moreover, infecting cervical cancer HeLa cells with NDV leads to an increase in IRE1α phosphorylation and XBP1s expression, thus activating the IRE1-JNK pathway and stimulating viral replication [74]. Another recent study reveals the expansion of the ER lumen and a significant increase in intracellular BiP expression in Quail Muscle (QM7) cells infected with the infectious bursal disease virus (IBDV). IBDV-induced ERS lead to the accumulation of lipid droplets (LDs), which do not play a significant role in IBDV replication [75], suggesting that ERS may not affect IBDV replication.

**Figure 1:**
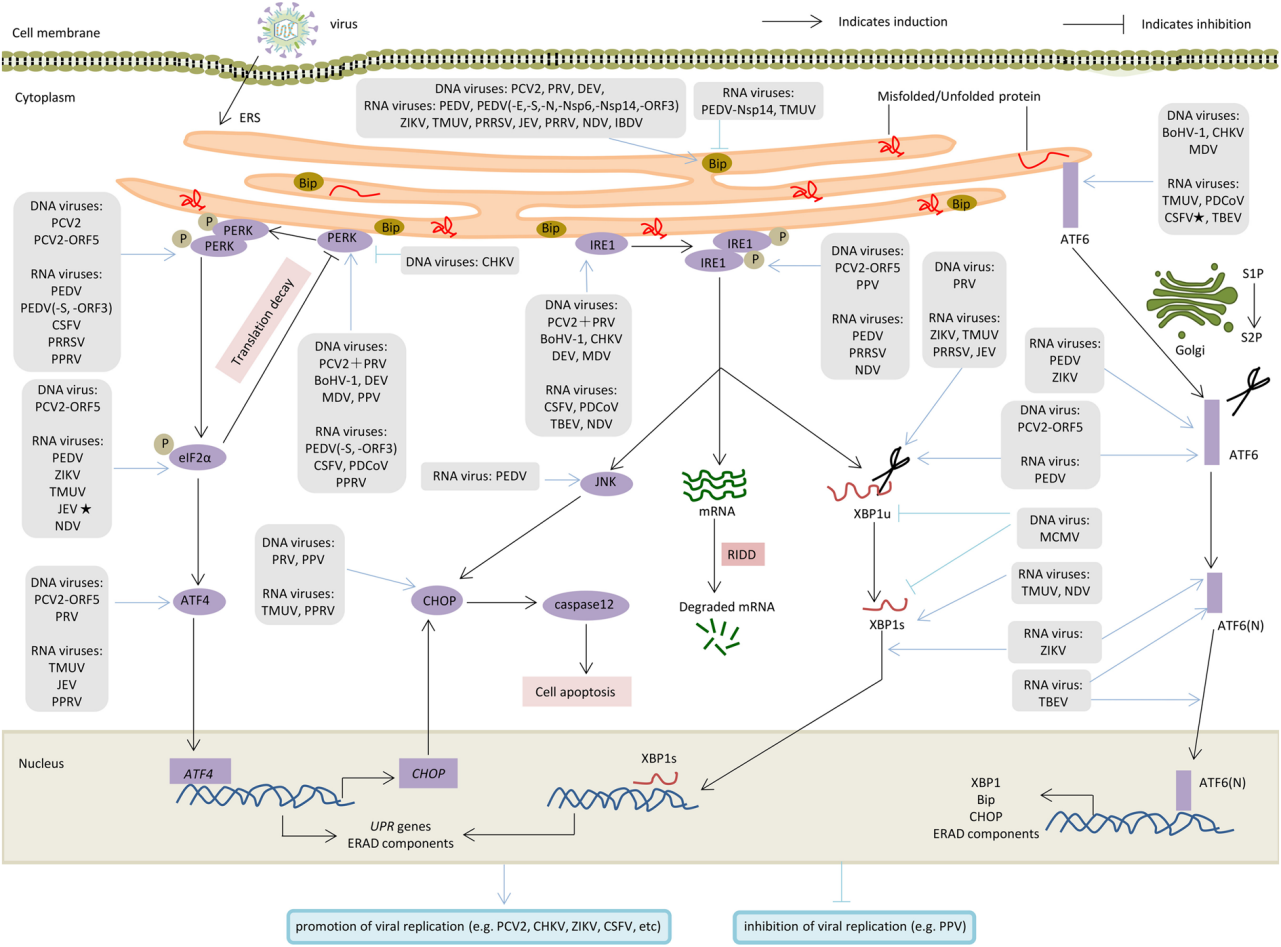
**Three UPR signalling pathways driven by ERS in response to animal viral infections**. Accumulating misfolded or unfolded proteins in the ER lumen cause ERS, followed by activation of the typical UPRs to relieve ERS. Dissociation from BiP leads to the activation of three ER transmembrane protein sensors: PERK, IRE1, and ATF6. For the PERK signalling pathway, PERK first undergoes phosphorylation and dimerization, followed by phosphorylation of eIF2α. p-eIF2α can extensively inhibit intracellular protein translation but can selectively enhance the expression of ATF4, which can activate CHOP expression and promote the expression of UPR genes and ERAD components. For the IRE1 signalling pathway, phosphorylation and dimerization of IRE1 not only lead to the excision of XBP1 mRNA to form the mature form of XBP1s, which translocates to the nucleus and promotes expression of the UPR genes and ERAD components but also selectively promote apoptosis through the JNK-caspase12 pathway or directly promotes mRNA degradation through RIDD. ATF6 translocates to the Golgi and is cleaved to form cleaved ATF6(N), which enters the nucleus and binds on the ERSE to promote the expression of BiP, XBP1, CHOP, and ERAD components. A substantial number of animal viruses induce ERS in infected host cells. The ERS-driven UPR either inhibits or promotes viral proliferation. Black and blue pointed arrows denote activation, and black and blue blunt-end arrows denote inhibition. A ★ in the figure represents differences in the molecular mechanisms and regulation of viral replication caused by the same animal viruses. For example, JEV infection with BV2 cells activates the PERK and IRE1 pathways (marked as this in the figure), but infection with SH-SY5Y cells only activates the PERK pathway. ST cells, immune and non-immune organs infected with CSFV activate the three UPR pathways (marked as this in the figure), but CSFV infection with PK-15 cells slightly inhibits the ATF6 pathway.

Collectively, these outcomes indicate that animal virus infections causing ERS are a common phenomenon and that different viruses are capable of selectively regulating UPR sensors during the infection of host cells. The replication of some viruses is shown to be restricted by the ERS, while others can hold the ERS hostage for replication to maintain a persistent infection (Figure 1). For example, PPRV and CHKV are attributed to different families and regulate different UPR pathways, which are suggested to promote viral replication. Interestingly, even viruses belonging to the same family may have different mechanisms for regulating UPR: ZIKV, TMUV, CSFV, and JEV belonging to the *Flaviviridae* family induce ERS after viral infection and promote viral replication. However, there are some differences in the triggered UPR pathways. Thus, the effect of ERS induced by different virus infections on viral replication and their mechanisms of action may differ. These disparities may be related to several factors, including cellular properties, viral specificity, and viral adaptations within host cells.

## Autophagy in animal viral infections

Cellular autophagy is a conserved activity in eukaryotic cells that degrades and recycles intracellular substrates. It is involved in a wide range of physiological and pathological processes to maintain intracellular homeostasis. There are three main types of autophagy: macroautophagy (hereafter referred to as autophagy), microautophagy, and chaperone-mediated autophagy. Among these, autophagy is the most studied [76, 77]. The process of autophagy usually involves the encapsulation of substrates, such as damaged organelles and unfolded or misfolded proteins, in the cytoplasm by a double-layered membrane vesicle structure (i.e. formation of an autophagosome). The autophagosome combines with the lysosome to form an autolysosome, leading to the degradation of its contents by various types of enzymes in the lysosome [78].

Under normal conditions, cells undergo a low level of constitutive basal autophagy. However, autophagic activity significantly increases when cells are exposed to unfavourable conditions such as stress, infection, or cancer. This results in the degradation of cytoplasmic macromolecules into metabolites for recycling by the cell [79] for the purpose of protective autophagy. Although autophagy typically has a positive effect, such as promoting stress relief and cell survival [80], it can also be hijacked by many viruses as a potential immune evasion mechanism. The molecules involved in autophagy-related signalling may be potential targets for preventing and controlling animal viral diseases, which has become one of the hot topics of interest to researchers currently.

Virus-host interactions often involve activating or inhibiting signalling molecules, and autophagy is no exception. Each step of autophagy is tightly regulated by a large number of highly conserved autophagy-related genes (ATGs). When viral infection stimulates autophagy, the mammalian target of rapamycin (mTOR) is inhibited. This inhibition results in a reduced phosphorylation of the ULK complex formed by Unc-51-like kinase 1 (ULK1), ULK2, ATG13, ATG101, and focal adhesion kinase family kinase-interacting protein of 200 KD (FIP200). The ULK complex is then transferred to the ER. Consequently, autophagy is initiated [81].

The ULK complex is responsible for recruiting the class III phosphatidylinositol-3-OH kinase (PI3K) complex, which consists of vacuolar protein sorting 34 (Vps34), Vps15, ATG14, and Beclin1. In the PI3K complex, Beclin1 phosphorylates the Vps34 to produce phosphatidylinositol-3-phosphate (PI3P), which recruits effector factors and promotes nucleation of autophagosomes [82–84]. The process of autophagosome elongation requires two ubiquitinated processing systems. First, the ATG12-ATG5-ATG16 complex is located on the outside of the autophagosome structure and is essential for autophagosome elongation. Second, the cytoplasmic class I microtubule-associated protein 1 light chain 3 (LC3-I) in the cytosol is recruited to the autophagosome membrane and conjugated with phosphatidylethanolamination to generate type II LC3 (LC3-II), thus participating in autophagosome formation [85]. Due to the elevation of LC3-II and its co-localisation with the autophagosome membrane, LC3-II-related assays have been widely used to detect autophagy [86].

In the final stage of autophagy, autophagosomes fuse with lysosomes to form autolysosomes. Within these autolysosomes, lysosomal enzymes degrade the contents, which allows for the recycling of biomolecules [85]. Although the process of degrading contents is inherently antiviral in nature, some viruses, particularly RNA viruses, have developed mechanisms to avoid, subvert, or co-opt the process to their advantage [87]. Several signalling pathways have been found to play a part in regulating autophagic activities. The most widely studied pathway that negatively regulates regulation is the class I PI3K/mTOR pathway. In contrast, the class III PI3K/Beclin1 pathway positively regulates autophagy [88, 89]. Furthermore, many factors, such as the tumour suppressor protein phosphatase and tensin homolog (PTEN) and adenosine monophosphate-activated protein kinase (AMPK), are also involved in regulating autophagy [90, 91]. As an upstream regulator of mTOR, activated AMPK can ultimately inhibit cellular protein synthesis by activating tuberous sclerosis complex 2 (TSC2) and inhibiting mTOR complex 1 (mTORC1) activity [90, 92, 93]. Inhibition of the phosphorylation activity of mTORC1, a negative regulator of autophagy, therefore induces autophagy [90, 93].

Increasing evidence show that many mammalian cells can mount autophagic responses during the development of viral infection. On one hand, host cells can activate the autophagy mechanism to fight against viral infections. However, on the other hand, certain viruses can hijack cellular autophagy to support their replication. In other words, autophagy can be used both as a mechanism for viral clearance and as a means of viral replication.

### DNA viruses associated with autophagy

The growing evidence in this field demonstrates that autophagy can be induced in animal virus infections. However, to counteract the antiviral effects of autophagy, many viruses have developed strategies to either evade, impair, or even enhance autophagy, allowing for more effective immune escape and persistent replication. Some DNA viruses-induced autophagy is disposed to play a positive role in promoting viral replication. Both PCV2 and PPV induce autophagy by activating the AMPK pathway. Equally, PCV2 also activates extracellular regulated protein kinases (ERK1/2) and TSC2 and inhibits mTOR signalling. For instance, PCV2 replication in PK-15 cells is enhanced via the AMPK/ERK/TSC2/mTOR signalling pathway, whereas PPV induces autophagy to promote viral replication by inhibiting mTORC1 [94, 95].

Both egg drop syndrome virus (EDSV) infection of duck embryo fibroblasts (DEF) cells and orf virus (ORFV) infection of ovine foetal turbinate (OFTu) cells down-regulate PI3K/AKT/mTOR to induce autophagy. This down-regulation promotes self-replication in host cells [96]. In OFTu cells, ORFV infection results in an increase in TSC2 phosphorylation and a decrease in mTOR phosphorylation. This process occurs through the suppression of the PI3K/AKT/mTOR signalling pathway and the activation of the ERK1/2/mTOR signalling pathway, inducing complete autophagy as the autophagosomes fuse with lysosomes in autophagic flux for viral replication [97, 98].

In contrast, certain DNA viruses, including African swine fever virus (ASFV), PRV, PCV2, PPV, EDSV, and ORFV, have the ability to enhance viral replication by inhibiting autophagy. ASFV infection inhibits autophagy by activating mTORC1 and significantly reduces cell numbers [99]. Additionally, during ASFV infection, the viral protein A179L encoded by ASFV is homologous to Bcl-2 and can interact with Beclin1 [100], thereby reducing the free state of Beclin1. This interaction suggests that the virus inhibits autophagy, which in turn promotes viral replication, i.e. autophagy plays an active antiviral role during ASFV infection. Furthermore, the periplasmic protein US3 of PRV can activate the AKT/mTOR pathway to inhibit autophagy, promoting PRV replication [101].

The combined processes of cellular autophagy, influenced by various DNA viruses, may have diverse effects on viral replication. Recent literature suggests that DNA viruses tend to utilise the autophagic mechanism to boost viral replication. This strategy could potentially help animal viruses evade the immune system.

### RNA viruses associated with autophagy

Even though viruses are evolutionarily limited in genome size, they can manipulate host cell processes, such as autophagy, by using multifunctional viral proteins, molecular mimicry of host components, and the inherent high mutagenicity of their RNA genomes. As such, this allows the virus to not only obtain nutrition but also to achieve immune evasion [102].

CSFV infection provokes the formation of LC3-I/LC3-II transition and ATG12-ATG5. These two ubiquitin-like conjugation systems are coupled to participate in the process of autophagosome elongation. Meanwhile, it has been observed that CSFV-infected PK-15 and 3D4/2 cells result in the increased expression of ATG5 and Beclin1, which triggers an autophagic response. This response ultimately leads to the enhanced replication and maturation of CSFV in the host cells [103]. Furthermore, dengue virus type 2 (DENV2) induces autophagy in human umbilical vein endothelial (HUVE) cells by inhibiting mTOR signalling molecules, which favours DENV2 replication [104].

Infection by NDV activates the AMPK signalling pathway while inhibiting mTORC1 activity and activating ULK1 [50, 105]. NDV HN and F proteins together induce autophagy through coordinated activation of the AMPK/mTORC1/ULK1 pathway and synergistically induce fusion of autophagosomes with lysosomes for subsequent degradation. However, the effect on viral replication is unknown [105]. PEDV Nsp6 protein, SADS-CoV, and canine distemper virus (CDV) N protein induce autophagy via the AKT/mTOR axis to promote viral replication [106–111]. There are also cases where virus-induced autophagy does not affect viral infection, such as equine herpesviruses 1 (EHV-1) [112].

ZIKV requires both mTORC1 and mTORC2 activation to regulate autophagy negatively and promote ZIKV replication [113]. It has been reported that MCMV and grass carp reovirus (GCRV) can activate the AKT/mTOR pathway to inhibit autophagy, promoting the replication of these viruses [114, 115]. Specifically, MCMV inhibits autophagy by activating the PI3K/AKT/mTOR pathway [114]. Compared with complete autophagy induced by SADS-CoV and CSFV, the NSP3 and NSP5 proteins of PRRSV, the P5 protein of rabies virus (RABV), and the 2C protein (non-structural protein) of foot-and-mouth disease virus (FMDV) can induce autophagosome formation without fusion with lysosomes, namely incomplete autophagy.

However, the method by which PRRSV NSP3 and NSP5 proteins create autophagosomes has not been specified. RABV P5 protein and FMDV 2C protein induced autophagy by binding to Beclin1, which enhanced viral replication of RABV and FMDV [116–118]. Research has shown that IBDV induces autophagic signalling in the late stage of infection, and interestingly, IBDV infection induces autophagosome-lysosome fusion without actively degrading its contents. Further studies have revealed that inhibition of fusion or lysosomal hydrolysis activity significantly inhibits viral replication. This inhibition suggests that IBDV likely utilises the low pH environment of acidic organelles to promote viral protein maturation and replication [119].

In contrast, Muscovy duck reovirus (MDRV) inhibits autophagy-lysosomal fusion, and SARS-CoV-2 inhibits autophagy-lysosomal degradation [120], although both promote the replication of the virus itself [121, 122]. Additionally, SARS-CoV-2 can activate AKT to inhibit autophagy and promote viral replication [120]. The effects of the interaction between autophagy and viruses are dependent on the cell and type of virus and are typically observed as a method of enhancing viral replication.

The autophagy mechanisms and their effects on viral replication may differ even when caused by the same virus. It is worth noting that the PEDV strain JS-2013 inhibits autophagy by activating the PI3K-AKT pathway, thus inhibiting its infection with Vero cells [108]. Overexpression of PEDV Nsp6 protein in intestinal porcine epithelial cell line-J2 (IPEC-J2) enhances the replication of PEDV strain YC2014 by inducing autophagy via inhibition of the PI3K/AKT/mTOR signalling pathway [109]. However, there is a discrepancy in the findings of research reports on the effects of PEDV on autophagy and their roles in modulating viral replication [108, 109]. These discrepancies may be attributed to differences in the strains and cells used in the studies. Both JEV strain SA14-14–2 infection in BHK-21 cells and JEV strain P3 infection in mammalian mouse brain activate autophagy to promote viral replication [123–125]. However, it has been reported that JEV strain P20778 infection of Neuro2a cells activates autophagy through ERS-driven XBP1 and ATF6 pathways, thus inhibiting the replication of JEV [13]. It is puzzling that JEV infection-induced autophagy has different effects on viral replication, which may be related to differences in viral strains, cell types, and signalling pathways that regulate autophagy.

**Figure 2:**
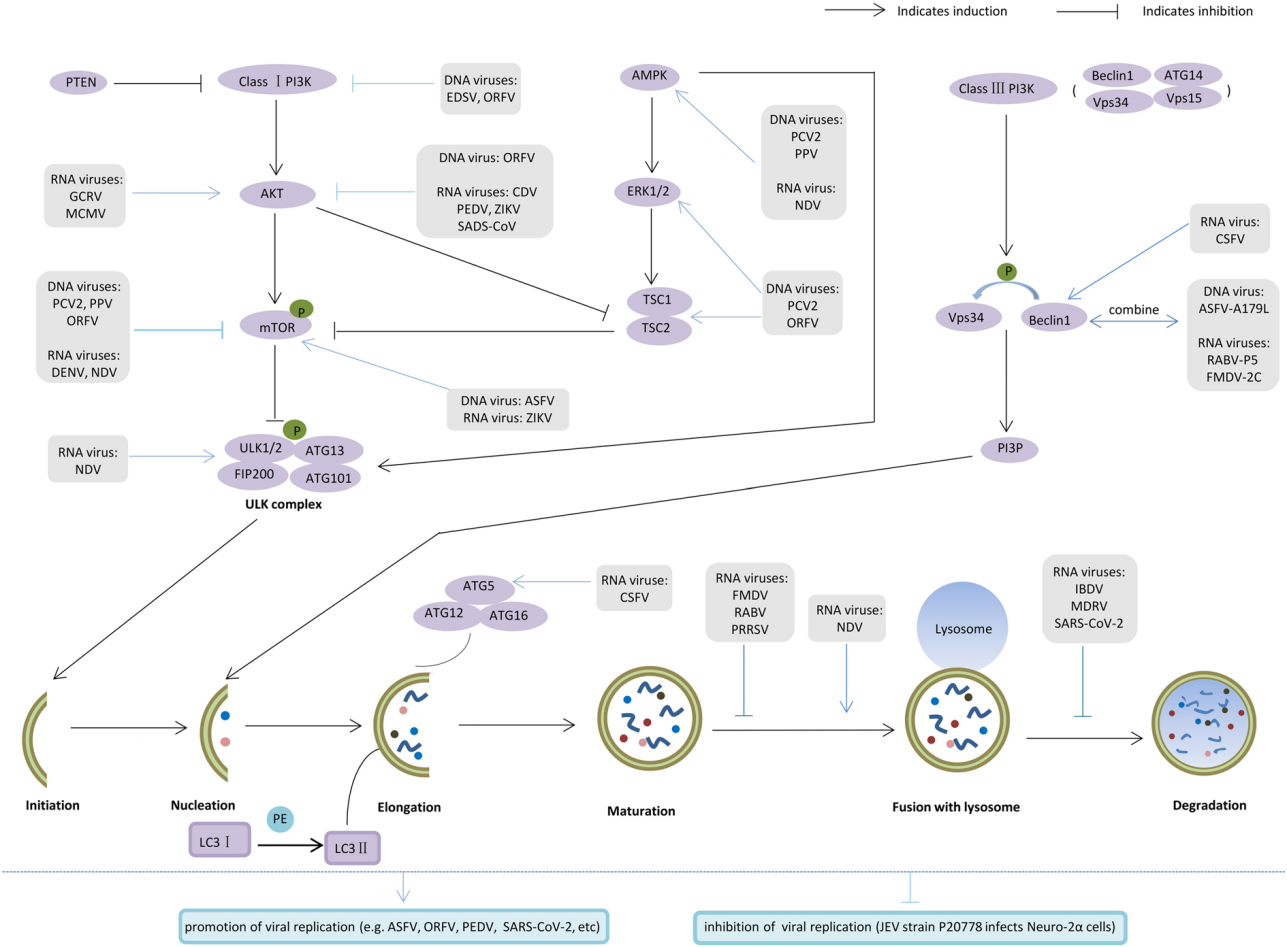
**Mechanisms by which animal viral infections modulate cellular autophagy.** Under stress conditions, class I PI3K-AKT-mTOR is the most common autophagy signalling pathway, and inhibition of this pathway facilitates the binding of ULK1/2 and dephosphorylated ATG13, ATG101, and FIP200, which form a complex that translocates to the ER and initiates autophagy, i.e., this pathway has a negative feedback effect on autophagy. In addition, AMPK can be indirectly or directly involved in regulating ULK complexes through the ERK1/2-TSC1/2-mTOR pathway. The ULK complex is responsible for recruiting the class III PI3K complex to regulate autophagy positively, and the class III PI3K complex mainly consists of Vps34, Vps15, ATG14 and Beclin1 (ATG6), and the Vps34 protein-activated by Beclin1 produces PI3P, thereby recruiting effector factors and promoting nucleation of autophagosomes. The formation of the ATG5-ATG12-ATG16 complex and the transition from LC3-I to LC3-II state play crucial roles in the process of autophagosome elongation and closure. After the extension and maturation stages, autophagosomes fuse with lysosomes to form autophagic lysosomes, with internal lysosomal enzymes that can degrade contents such as misfolded or unfolded proteins and organelles. Black and blue pointed arrows denote activation, and black and blue blunt-end arrows denote inhibition.

Recent research, therefore, implies that animal viral infections often trigger autophagy and that different viruses selectively regulate autophagy during infection of host cells. Additionally, most animal viruses are currently reported to hold autophagy hostage to maintain their replication and infection, although there are a limited number of viruses where the replication is restricted by autophagy (Figure 2).

## ERS-mediated autophagy in animal viral infections

### ERS-mediated autophagy

Multiple stimuli can disrupt ER homeostasis in eukaryotic cells, resulting in the induction of ERS. These cells then use a highly conserved mechanism that activates three UPR pathways (PERK, IRE1 and ATF6) to reduce ERS and restore ER homeostasis [126]. Autophagy, an evolutionarily conserved process of degradation and recycling in eukaryotic cells, is thought to facilitate cell survival and protect the cell from unfavourable conditions such as nutrient deprivation and pathogen infection [127]. However, excessive or uncontrolled autophagy can induce autophagy-dependent cell death [128]. There is mounting evidence to suggest that ERS-driven activation of the UPR pathways during certain virus infections is an important trigger of autophagy [129, 130].

ERS-mediated cellular autophagy was first reported in 2006 [131]. As research has progressed, it has been established that the above three UPR pathways can induce autophagy. To minimise the detrimental effects of ERS, the host initiates protective autophagy to counteract ERS and sustain the balance of ER homeostasis [132]. Elucidating the molecular mechanism of ERS-mediated autophagy could enhance our understanding of the pathogenesis of certain diseases. Additionally, this knowledge could be utilized for disease prevention and control by intervening and regulating the target genes or proteins involved.

Following viral infection of host cells, the accumulation of misfolded or unfolded proteins in the ER can lead to ERS and induce UPR. This process then triggers autophagy to defend against viral infection. In the IRE1 pathway of the UPR, IRE1 undergoes dimerisation and autophosphorylation before binding to TNF receptor-associated factor 2 (TRAF2) and apoptosis signal-regulating kinase (ASK1) to form a complex that activates JNK downstream. Activated JNK promotes the phosphorylation of Bcl-2, which disrupts the association of Beclin1 and Bcl-2, leaving Beclin1 in a free state to bind to Vps34, Vps15 and ATG14. The binding subsequently forms the class III PI3K complex, which promotes membrane nucleation for autophagy [133, 134]. In addition, XBP1 mediated by IRE1 also triggers autophagy through transcriptional activation of Beclin1 [134]. Similarly, in the PERK pathway, phosphorylation of eIF2α selectively promotes ATF4 translation.

On the one hand, the ATF4 protein not only directly promotes the production of ATG12 and ATG16 but also drives the activation of CHOP to stimulate the production of ATG5, which then forms the ATG5-ATG12-ATG16 complex together with ATG12 and ATG1. This complex plays a key role in autophagosome extension [134, 135]. In the ATF6 pathway, upon ERS, ATF6 cleaved by S1P and S2P in the Golgi can up-regulate the expression of death-associated protein kinase 1 (DAPK1), which phosphorylates Beclin1. The cleaved ATF6 also induces XBP1 and CHOP expression to regulate autophagy or directly up-regulates the transcription of autophagy-related genes such as *LC3*, *ATG12* and *ATG5* [136].

In addition to the three UPR pathways mentioned above that can mediate autophagy in viral infection, the release of Ca^2+^ contained in the ER can also serve as a regulator for autophagy. The inositol trisphosphate receptor IP3R, located on the ER, can promote the release of Ca^2+^ from the ER lumen into the cytoplasm and activate the regulatory calmodulin-dependent protein kinase-II (CaMKKII) and DAPK. CaMKKII promotes the generation of the ULK1 complex via the AMPK-mTOR pathway. In contrast, the activation of DAPK promotes the phosphorylation of Beclin1. These two regulatory modalities of Ca^2+^ play an important role in inducing autophagy [133, 134, 137].

During ERS-induced autophagy, the UPR regulates autophagy via the IRE1α, PERK, ATF6 and Ca^2+^ pathways, in which CHOP plays critical roles. Increasing evidence suggests that the ERS-driven UPRs unfolded protein responses play critical roles in inducing and regulating autophagic pathways [82].

### Roles and mechanisms of ERS-mediated autophagy in animal virus replication

After viral infection, the host instinctively triggers protective strategies to control the viral infection, such as ERS-mediated autophagy as a cellular adaptive mechanism. For instance, although JEV infection activates three UPR pathways in neuronal cells, it inhibits JEV replication by only activating autophagy through the ATF6 sensor and XBP1 [13]. However, to achieve persistent replication in the host, some viruses evolve a specific strategy via the ERS-mediated autophagy pathway. Regarding nucleic acid types, there are fewer reports in the literature of DNA viruses that can promote viral replication through ERS-mediated autophagy. DEV infection activates ERS, while inhibition of PERK and IRE1 expression reduces the transformation of LC3I to LC3II in DEV-infected DEF cells and inhibits DEV replication [44]. This outcome suggests that DEV positively regulates cellular autophagy via the PERK-eIF2α and IRE1-XBP1 pathways, contributing to viral replication. The phosphorylation of PERK and eIF2α activated by PCV2 ORF5 protein induces autophagy in PK-15 cells, and PCV2 replication is promoted through the PERK-eIF2α-ATF4 and AMPK-ERK1/2-mTOR pathway [82].

Compared to DNA viruses, RNA viruses that induce ERS-mediated autophagy have been reported more frequently. These include viruses that mediate autophagy through the three pathways of the UPR. The study found that the IRE1-JNK-Beclin 1 signalling pathway, PERK-eIF2α, and ATF6 pathways are essential in SADS-CoV-induced autophagy. The study further explored whether autophagy induced by the ERS sensor IRE1 but not PERK-eIF2α and ATF6 promote SADS-CoV replication [110].

Particular viruses mediate autophagy through two pathways of the UPR, such as PEDV, DENV, CSFV, and NDV. Furthermore, recombinant NDV (rL-RVG) was found to enhance autophagic activity and viral replication through the PERK and IRE1 pathways [14, 84, 138, 139]. To promote viral replication, PEDV induces autophagy through the PERK-eIF2α and IER1-JNK pathways in Vero cells. It has been found that the PEDV ORF3 protein increases BiP expression and activates the PERK-eIF2α signalling pathway for the promotion of autophagy [14, 57]. During DENV infection, the PERK-eIF2α-ATF4-ATG12 and IRE1α-JNK-Beclin1 signalling pathways increased autophagy and viral load. However, the ATF6 pathway appears not to influence autophagy and viral replication.

Additionally, Beclin1 plays a key role in autophagy activation and activated JNK phosphorylates Bcl-2 and dissociates it from Beclin1, which is the main signalling pathway that induces autophagy and thus promotes DENV infection. This study also suggests that treatment with JNK inhibitors reduces DENV titers [138], implying that JNK is a potential target for combating DENV. The previous studies conducted by our research group have also shown that CSFV infection induces ERS-mediated autophagy for effective viral infection in vitro and in vivo. Moreover, further studies confirm that CSFV infection induces complete autophagy by activating the PERK-eIF2α-ATF4-CHOP and IRE1/BiP pathways to promote viral replication in cultured cells [15, 139].

Cells infected with NDV or transfection with NDV NP or P proteins activate PERK and ATF6-dependent autophagy to maintain NDV replication [140]. In a separate study, infection with rL-RVG stably expresses RABV glycoproteins by inserting the RABV glycoprotein gene between the *P* and *M* genes of the NDV. This insertion induced autophagy through the PERK-eIF2α-Beclin1 and IRE1-JNK-CHOP signalling pathways, thus promoting viral replication [141]. It has also been found that the Seneca valley virus (SVV), an important emerging porcine virus, promotes autophagy and SVV production by inducing the PERK and ATF6 pathways of UPR upon its infection [142]. To this point, most of the literature that reports animal viruses-induced autophagy via a single UPR pathway is related to the PERK pathway, which is the preferred pathway activated in response to viral infection. During infection with PPRV and bluetongue virus (BTV), PERK and eIF2α phosphorylation levels are increased, respectively, as well as LC3II levels.

Conversely, inhibition of PERK or knockdown of eIF2α not only reduces LC3II levels but also decreases the expression of PPRV N and C proteins and BTV VP2 protein. This finding suggests that PPRV and BTV-induced activation of the PERK-eIF2α pathway positively regulates autophagy and favours viral replication [143, 144]. During FMDV infection, VP2 protein promotes viral infection by activating the eIF2α-ATF4 pathway, thereby inhibiting the AKT-mTOR pathway to trigger autophagy [145]. Similarly, the red grouper nervous necrosis virus (RGNNV) induces autophagy by activating eIF2α phosphorylation and inhibiting mTOR phosphorylation; the enhanced autophagy contributes to RGNNV replication [146]. PRRSV infection induces ERS and activates the PERK and IRE1 rather than ATF6 signalling pathways to promote viral replication. However, the decreased Beclin1 and LC3-II only occur after PERK knockdown, further suggesting PERK-dependent autophagy in PRRSV infection [66]. In addition, PRRSV Nsp2 can interact with BiP and stromal interaction molecule 1 (STIM1) to induce autophagy [147]. PRRSV infection also leads to dysregulation of Ca^2+^ homeostasis, which is further exploited to promote viral replication through CaMKKII-AMPK-mTOR signalling-mediated autophagy [137].

**Figure 3:**
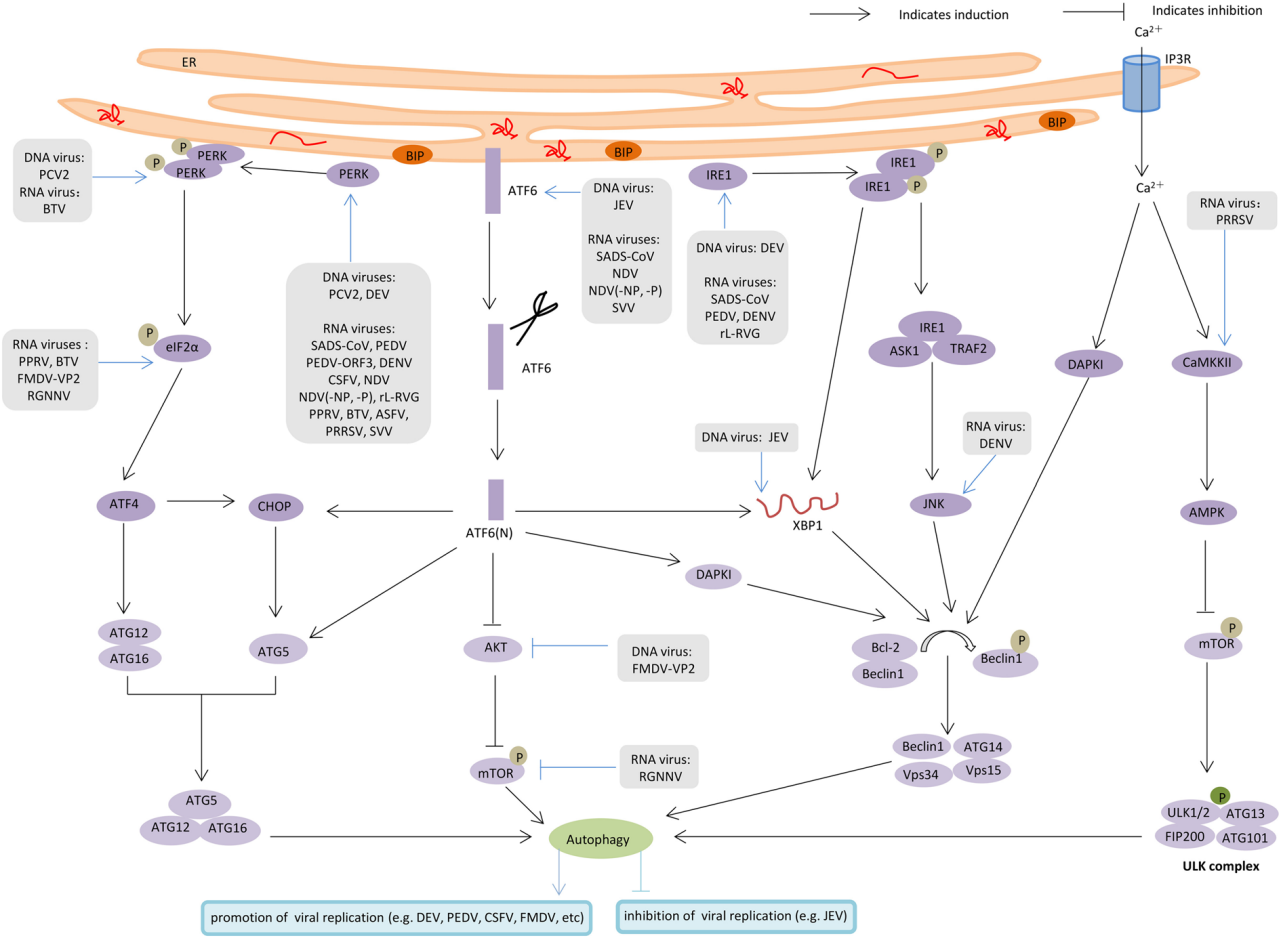
**Mechanisms of ERS-mediated autophagy in animal virus infections.** Some animal viruses induce ERS to regulate the activation of autophagy through three UPR signalling pathways: ATF6(N) is formed after ATF6 cleavage, ATF6(N) induces autophagosome formation through CHOP, or by directly regulating ATG5 transcription, or by negatively regulating the AKT-mTOR pathway, or by activating the DAPK1-Beclin1 pathway. The activated IRE1 forms complexes with TRAF2 and ASK1, activating the JNK downstream pathway and then causing Bcl-2 phosphorylation, thereby releasing free Beclin1. In addition, XBP1 also triggers transcriptional activation of Beclin1, resulting in the formation of the Vsp15-Vps34-Beclin1-ATG14 complex to promote vesicle nucleation. Activated PERK regulates the transcription of ATG12 and ATG16 through ATF4, which activates CHOP to induce transcription of ATG5. The formation of the ATG5-ATG12-ATG16 complex engages in the process of autophagosome elongation. Additionally, the ERS state induces Ca^2+^ imbalance, and Ca^2+^ release from the ER lumen via IP3R activates the CaMK-AMPK-mTOR pathway, which promotes the formation of the ULK1 complex to trigger autophagy. Ca^2+^ release also activates DAPK1 to promote Beclin-1 phosphorylation, thus promoting autophagy. Black and blue pointed arrows denote activation, and black and blue blunt-end arrows denote inhibition.

In summary, although ERS and autophagy normally promote cell survival and antiviral activity, many viruses have evolved specific strategies to regulate ERS-mediated autophagy to maintain efficient replication in host cells (Figure 3). Understanding these molecular mechanisms can help identify drug targets and develop new antiviral strategies that target the ERS-mediated autophagy pathway. For example, in the future, it may be possible to use gene editing techniques (e.g., the CRISPR/Cas9 system) to modify the genes of key target molecules in the ERS-mediated autophagy pathway, thereby enhancing the body’s antiviral capacity.

### Crosstalk between ERS-mediated autophagy and ER-phagy

ER-phagy is a form of selective autophagy that uses the ER as a specific substrate. There are at least two types of ER-phagy: macro and micro [148]. ER-phagy is primarily mediated by specific ER-phagy receptors that connect the ER and autophagosomes. These receptors predominantly include family with sequence similarity 134 member B (FAM134B), translocation protein SEC62 (SEC62), reticulon 3 (RTN3), cell cycle progression 1 (CCPG1), atlastin 3 (ATL3), and testis expressed protein 264 (TEX264). The receptors act by recruiting degraded cargo on the lumen side of the ER and then binding to the autophagy machinery on the cytosolic side of the ER, transporting the cargo for lysosomal degradation [149–151].

In addition to ERS, autophagy, and ER-mediated autophagy, ER-phagy can also evolve via the mammalian cells to circumvent ER imbalance induced by misfolded or unfolded proteins. It can occur under normal physiological conditions and when cells are subjected to environmental changes such as starvation, UPR, and toxin stimulation. The primary role of ER-phagy is to repair ER dysfunction and maintain ER homeostasis [148, 149, 152]. The autophagosome formation process of ER-phagy is very similar to that of autophagy, the difference being that ER-phagy achieves substrate selectivity through the ER-phagy receptor [148]. A small amount of literature has been published linking ER-phagy in some animal viral infections.

On the one hand, ER-phagy inhibits the proliferation of certain animal viruses in host cells. However, on the other hand, some animal viruses can develop specific strategies to regulate ER-phagy and promote the release and spread of viral offspring by hijacking the host’s ER-phagy pathway. For example, FAM134B-mediated ER-phagy has been evidenced to inhibit the replication of Ebola virus (EBOV) strains Makona and Mayinga in Vero-E6 cells. It has also been shown to play a negative regulatory role in replicating DENV, ZIKV and SARS-CoV-2 [153–155]. Both DENV and ZIKV can use the NS3 proteases to directly cleave FAM134B at a single site within their reticular homology domain (RHD), which in turn inhibits ER-phagy and thus promotes viral replication [154]. SARS-CoV-2 damages ER-phagy by hijacking FAM134B and ATL3 into p62 condensates, increasing viral replication [155].

Additionally, RTN3-mediated ER-phagy negatively regulates viral replication by interfering with the NS4B protein of the hepatitis C virus (HCV) [156]. The association of these viruses with ER-phagy can be seen specifically in another review [157]. In addition to regulating animal viral replication through the ER-phagy pathway alone, research has shown that SEC62-mediated ER-phagy can promote FMDV clearance by activating IRE1α-JNK pathway-mediated autophagy and delivering autophagosomes to lysosomes [158]. This finding demonstrates the role that ER-phagy plays in the activation of ERS-mediated autophagy. Moreover, despite the importance of such a role, there appears to be little research on the interaction between ERS-mediated autophagy and ER-phagy and their potential specific mechanisms in animal virus infections. Therefore, further exploration and clarification of the interaction between the two is necessary. Addressing these issues will provide new insights into the replication and pathogenesis of animal viruses.

## Targeted therapies and strategies based on the ERS-mediated autophagy pathways and their applications in animal diseases

As previously discussed, many viruses have evolved mechanisms to hold ERS-mediated autophagy hostage to maintain viral infection in host cells. Given the crucial role ERS-mediated autophagy plays in the process of animal virus infection and replication, the development of targeted therapies and strategies based on ERS-mediated autophagy pathways (such as antiviral drugs that regulate the UPR or autophagy signalling molecules) represents a promising method for preventing and treating animal diseases. However, to date, reports related to this topic remain limited.

Inhibiting the key targets of ERS, such as BiP, PERK, IRE1, and ATF6, through particular drugs or technical means can positively affect anti-animal viruses. For instance, BiP is an important host factor that is the marker of ERS and targeting BiP with specific drugs can potentially reduce viral replication. Subtilase cytotoxin (SubAB, a BiP lysate) can lead to a 10- to 100-fold reduction in infectious DENV release. One study found that in the absence of BiP, SubAB does not affect normal RNA replication by DENV but rather blocks the formation of intracellular DENV viral particles and alters antigen levels of DENV [159]. Treating human monocytes with VER-155008 (WER, a known inhibitor of BiP) before DENV infection can decrease the expression of DENV envelope proteins. Such a strategy can be used to reduce DENV infection temporarily [160]. An important pathogenic factor for DENV is non-structural protein 1 (NS1), which is required for viral replication. Furthermore, ivermectin blocks the nuclear transport of transcription factors required for UPR, thereby impairing BiP up-regulation and NS1 secretion [161]. This impairment thereby alleviates the pathogenicity of DENV.

Likewise, SARS-CoV-2 uses the host receptor angiotensin-converting enzyme 2 (ACE2) for viral invasion. BiP, an important host co-factor for SARS-CoV-2 entry and infection, can form a complex with SARS-Cov-2-Spike protein (SARS-CoV-2-S) and ACE2 to help viral infection. Knockdown of BiP in VeroE6 cells and treatment with a humanised monoclonal antibody hMAb159 (selected for its ability to endocytosise BiP and its safe clinical characteristics in preclinical models) both significantly reduce cell surface BiP and ACE2 expression. Additionally, hMAb159 reduces SARS-CoV-2-S-driven viral entry and infection in vitro [162]. Furthermore, YUM70, a small molecule inhibitor of BiP, has been found to effectively block the entry and infection of SARS-CoV-2 mediated by either the original or mutant spike protein both in vitro and in vivo. YUM70 not only reduces SARS-CoV-2 infection but also inhibits the production of viral proteins after SARS-CoV-2 infection without affecting cell viability in vitro [163].

Currently, most of the methods of ERS-mediated autophagy-targeted inhibition of animal viral replication are based on the interference technology of ERS-related UPR signalling pathways and the use of inhibitor drugs. The antiviral potential for inhibiting the PERK and IRE1 pathways has been demonstrated in certain animal viruses such as BoHV-1, DEV, and PEDV. The viral titres of these three viruses are significantly reduced by siRNA and pretreatment cells with GSK2606414 and 4μ8C or STF-083010, respectively, thus reducing the expression of PERK and IRE1 [14, 39, 44]. Additionally, CHKV replication was inhibited by using 3-ethoxy-5,6-dibromosalicylaldehyde and AEBSF to inhibit the IRE1 and ATF6 pathways [40].

Similarly, inhibition of the PERK and ATF6 pathways also provides strategies to reduce certain animal viral infections, such as NDV and SVV. Research has shown that siRNA silencing of PERK or ATF6 can inhibit viral replication in NDV-infected human non-small-cell lung cancer (NSCLC) cell line A549 and SVV-infected BHK-21 cells [140, 142]. It has also been reported that inhibiting a single UPR pathway can negatively regulate viral replication, for instance, SADS-CoV, PPRV, FMDV, and BTV. Since three UPR pathways can be activated after SADS-CoV infection, only knockout (siRNA- IRE1) or inhibition (4μ8C) of IRE1 significantly reduces SADS-CoV N protein levels and viral load in cell culture supernatants [110].

In the PERK pathway, both GSK treatment and gene silencing of eIF2a lead to a decrease in the expression of PPRV N protein and BTV VP2 protein [143, 144]. The production of PPRV N protein and FMDV is reduced in cells with ATF4 knockdown, suggesting that inhibiting the eIF2a-ATF4 pathway may reduce PPRV and FMDV replication [144, 145]. However, unlike knockdown techniques, overexpression of CHOP protein can block PPV replication in PK-15 cells [46]. The ATF6 pathway, treated with siRNA-ATF6 and AEBSF, can significantly inhibit PDCoV replication [64]. This outcome suggests that the key ERS targets could potentially be used in developing antiviral treatments in the future.

Increasing evidence suggests that specific drugs targeting autophagy-related signalling molecules may be promising in preventing anti-animal viruses. For instance, 3-methyladenine (3-MA, PI3K inhibitor) treatment can induce autophagy, thereby reducing viral titers of EDSV, ORFV, CSFV, and GCRV [96, 98, 103, 115]. Autophagy induction reduces the spread of SARS-CoV-2 in primary human lung cells and intestinal organoids by targeting the autophagy pathway with the selective AKT inhibitor MK-2206 and the Beclin1-stabilising anthelmintic niclosamide [164]. Moreover, enhancing AKT-mTOR activity by insulin can decrease GCRV VP7 protein and viral titers of GCRV [115]. Inhibition of mTOR kinase by Torin1 or rapamycin (RAPA) leads to decreased ZIKV protein expression and progeny production [113]. Chloroquine (CQ), which inhibits the fusion process of autophagy with lysosomes, also inhibits autophagy and reduces viral production of EDSV, GCRV, and MCMV [96, 114, 115]. Interestingly, traditional Chinese medicine has also been studied in relation to anti-animal viral infections. Both tetrandrine (TET) and veratrolamide (VAM) are extracted from traditional Chinese medicine, and both have been found to block macropinocytosis by inhibiting the PI3K/AKT pathway, thereby effectively inhibiting ASFV and PEDV, respectively [165, 166]. In addition, Class I PI3K-specific inhibitors LY294002 exhibited similar antiviral activity to ASFV and PEDV as TET and VEM [165, 166], suggesting that TET, VEM, and LY294002 have the potential to be broad-spectrum antiviral agents against the PI3K/AKT pathway.

The use of certain technical methods to block signalling molecules targeted by autophagy also holds potential in combatting animal viruses. Knockout of AMPK using CRISPR/Cas9 can result in a reduction in PPV DNA copies [94]. The inhibition of autophagy by RNA interference targeting ATG7 can reduce the yield of EDSV progeny [96], and inhibition of autophagy with specific shRNAs targeting Beclin1 and LC3B can reduce CSFV replication [103]. siRNA-mTORC1 and siRNA-mTORC2 inhibit ZIKV replication, although the degree of siRNA-mTORC2 inhibition is less obvious [113].

Furthermore, microRNA (miRNA), a small, non-coding RNA, can play an important role in host response to pathogen infection by regulating their target gene expression after transcription [167, 168]. BDBV infection up-regulates the expression of bta-miR-2904 (miR-2904) in MDBK cells, miR-2904 inhibits autophagy in MDBK cells through ATG13, and overexpression of miR-2904 inhibits the replication of BVDV NADL strains [167]. Additionally, ARV infection significantly increases the expression of Gga-miR-30c-5p in DF-1 cells, which inhibits viral replication by targeting ATG5 to inhibit ARV-induced autophagy [168]. These results demonstrate that overexpression of miRNA could effectively combat animal virus infections such as anti-BDBV and ARV. Based on these findings, it can be inferred that targeting key autophagy pathways holds promise for developing treatments against animal viruses.

ERS-mediated autophagy plays a crucial role in viral replication. Therefore, targeting ERS-mediated autophagy could be more effective in influencing viral replication, but there are scarce reports regarding this approach. Our group's previous study showed that TUDCA pretreatment further reduces the 3-MA-reduced CSFV replication. In contrast, TG pretreatment effectively increases the 3-MA reduced CSFV replication. These results indicate that 3-MA inhibits autophagy and thus reduces CSFV replication. Additionally, it can also be regulated by ERS [15]. This regulation suggests that for animal viruses that can trigger ERS-mediated autophagy, the combined targeting of ERS and autophagy may have the potential for more effective treatment of animal viral infections.

In summary, modulating the UPR and autophagy pathways provides a new perspective for antiviral approaches to ERS-mediated autophagy induced by animal viruses. These studies suggest that targeting the ERS and autophagy pathways may be helpful in the development of antiviral drugs that inhibit animal viral replication. However, improving the understanding and effectiveness of antiviral drugs still requires more research and practical applications.

We look forward to more relevant literature on the subject in the future.

## Conclusion

The ERS-mediated autophagy pathway plays a crucial role in regulating intracellular homeostasis and is closely linked to the infection and pathogenesis of several animal viruses. An increasing number of studies have established that many viruses are capable of using the ERS-mediated autophagy pathway to evade the immune system, thereby enabling viral replication and causing infection in the host. However, the effect of ERS-mediated autophagy on viral replication, infection, and its mechanisms of action varies among different viruses (even viruses belonging to the same family). For instance, CSFV, DENV, and JEV are all members of the *Flaviviridae* family. CSFV infection induces complete autophagy, and DENV mediates autophagy by activating the PERK and IRE1 signalling pathways, all of which can be used to promote viral replication. In contrast, JEV induces autophagy mediated by the ATF6 sensor and XBP1, the main target of IRE1, which negatively regulates its replication. In summary, regulating cellular ERS-mediated autophagy pathways during animal viral infections may be specific to the virus and play a critical role in enabling immune evasion and the persistence of viral replication through various mechanisms.

This paper has reviewed the latest research progress on the effects of ERS-mediated autophagy on animal viral infections and their molecular mechanisms. The paper has clarified the significant role of the ERS-mediated autophagy pathways in studying the pathogenic mechanisms of animal virus infections. The role of the ERS-mediated autophagy pathways positions a clear theoretical foundation for an in-depth understanding of the antiviral mechanisms of the hosts and the pathogenic mechanisms of animal viruses. Moreover, it reveals the collaborative relationship between animal viruses and hosts, thus contributing to finding suitable targets and developing targeted strategies (e.g. anti-viral drugs and vaccines) to prevent and treat animal viruses.

It has been shown that many viruses can regulate the ERS-mediated autophagy pathway to maintain their replication in host cells. However, the specific patterns and underlying molecular mechanisms of virus types that can mediate autophagy through specific ERS pathways, resulting in either inhibition or facilitation of viral proliferation, require further in-depth study and clarification.

Moreover, it is crucial to determine and clarify which viral proteins play key roles in the virus-induced ERS-mediated autophagy pathway and their underlying molecular mechanisms. It is also important to identify whether other interacted pathways, for example, ER-phagy, apoptosis, pyroptosis, and innate immunity, are associated with the roles and mechanisms of ERS-mediated autophagy in animal virus infections. Further explanation is needed for all these issues. It is essential to clarify these molecular mechanisms to identify new drug targets against the ERS-mediated autophagy signalling molecules. In doing so, there is the potential for the future development of new anti-viral therapies or strategies targeting the ERS-mediated autophagy pathway.

## Data Availability

Any materials utilised in this review are publicly accessible in the references.

## Abbreviations

ATF4: activating transcription factor 4
ATF6: activating transcription factor 6
ATL3: atlastin GTPase 3
AMPK: adenosine monophosphate-activated protein kinase
ASFV: African swine fever virus
ASK1: apoptosis signal-regulating kinase 1
BiP: immunoglobulin heavy chain binding protein
BoHV-1: bovine herpesvirus 1
BTV: bluetongue virus
CaMKKII: calmodulin-dependent protein kinase-II
Caspase12: cysteinyl aspartate specific proteinase 12
CCPG1: cell cycle progression 1
CDV: canine distemper virus
CHKV: Chikungunya virus
CHOP: C/EBP homologous protein
CSFV: classical swine fever virus
DAPK1: death-associated protein kinase 1
DEV: Duck enteritis virus
DENV: dengue virus
EBOV: Ebola virus
EDSV: egg drop syndrome virus
EHV-1: equine herpesviruses 1
eIF2α: eukaryotic initiation factor 2α
ER: endoplasmic reticulum
ERAD: ER-associated degradation
ERS: endoplasmic reticulum stress
FAM134B: family with sequence similarity 134 member B
FMDV: foot-and-mouth disease virus
GCRV: grass carp reovirus
GRP78: glucose-regulated protein 78
HCV: hepatitis C virus
IBDV: infectious bursal disease virus
IRE1: inositol-requiring enzyme 1
JEV: Japanese encephalitis virus
JNK: C-Jun N-terminal kinase
LC3: microtubule-associated protein light chain 3
LDSV: lumpy skin disease virus
MCMV: mouse cytomegalovirus
MDV: Marek’s disease virus
MDRV: muscovy duck reovirus
mTOR: mammalian target of rapamycin
NDV: Newcastle disease virus
ORFV: orf virus
PCV2: porcine circovirus 2
PEDV: porcine epidemic diarrhoea virus
PERK: protein kinase RNA-activated (PKR)-like ER resident kinase
PI3K: phosphatidylinositol-3-OH kinase
PI3P: phosphotidylinositol-3-phosphate
PDCoV: porcine deltacoronavirus
PPV: porcine parvovirus
PPRV: peste des petits ruminants virus
PRV: pseudorabies virus
PRRSV: porcine reproductive and respiratory syndrome virus
PTEN: phosphatase and tensin homolog
RABV: rabies virus
RGNNV: red grouper nervous necrosis virus
RHD: reticular homology domain
RIDD: regulated IRE1-dependent decay
RTN3: reticulon 3
rL-RVG: recombinant NDV
SADS-CoV: porcine acute diarrhoea syndrome coronavirus
SARS-CoV-2: severe acute respiratory syndrome coronavirus 2
SEC62: SEC62 homolog
SVV: Seneca valley virus
TBEV: tick-borne encephalitis virus
TEX264: testis expressed 264
TMUV: Tambusu virus
TRAF2: TNF Receptor-associated factor 2
TSC: tuberous sclerosis complex
ULK1: Unc-51-like kinase 1
UPR: unfolded protein response
Vps34: vacuolar protein sorting 34
XBP-1: X-box binding protein 1
ZIKV: Zika virus
